# Bacteremia and Urinary Tract Infection Caused by* Chromobacterium violaceum*: Case Reports from a Tertiary Care Hospital in Kathmandu, Nepal

**DOI:** 10.1155/2017/7929671

**Published:** 2017-04-05

**Authors:** Narayan Dutt Pant, Subhash Prasad Acharya, Raju Bhandari, Uday Narayan Yadav, Dil Bahadur Saru, Manisha Sharma

**Affiliations:** ^1^Department of Microbiology, Grande International Hospital, Dhapasi, Kathmandu, Nepal; ^2^Department of Critical Care Medicine, Grande International Hospital, Dhapasi, Kathmandu, Nepal; ^3^Department of Microbiology, GoldenGate International College, Battisputali, Kathmandu, Nepal; ^4^Forum for Health Research and Development, Dharan, Nepal; ^5^Department of Pathology, Grande International Hospital, Dhapasi, Kathmandu, Nepal; ^6^Department of Microbiology, Kathmandu Medical College, Kathmandu, Nepal

## Abstract

*Chromobacterium violaceum *is ubiquitous in the environment of tropical and subtropical regions but the infections caused by this organism are rare and the urinary tract infections caused by it are even rarer. Due to the propensity for hematogenous spread leading to fatal sepsis, the infections caused by* Chromobacterium violaceum* have high mortality rate (65–80%) with death occurring in as less as one week of acquiring infection. So, prompt proper treatment is necessary for successful treatment of the infections but, due to the rarity of the infections caused by the organism, there is limited awareness among the clinicians regarding the infections caused by this organism. Here, we reported a case of urinary tract infection caused by* Chromobacterium violaceum* in a 84-year-old male, who was a kidney patient, and another case of bacteremia caused by the same bacterium in a road traffic accident patient (22-year-old male), both of which were managed with the timely suitable treatment.

## 1. Introduction 

*Chromobacterium violaceum* is ubiquitously present in the environment of tropical and subtropical regions [1]. It is a normal flora of water and soil [1]. Human infections caused by this organism are very rare, due to which there is limited awareness about the infections caused by the bacterium [2]. Till 2007, around 150 cases of human infections have been reported worldwide [1] and recently four cases of infections caused by* Chromobacterium violaceum* have been reported from Nepal [1, 3–5]. In general,* Chromobacterium violaceum *is involved in causing fatal cases of sepsis, visceral abscesses, and skin and soft tissue infections with mortality rate up to 65–80% [3, 6]. But urinary tract infections caused by this organism are extremely rare [7]. In this study, we reported two cases of infections caused by* Chromobacterium violaceum*.

## 2. Case 1 (Urinary Tract Infection)

An 84-year-old chronic kidney disease patient attended the outpatient department of a hospital in Kathmandu, Nepal, with chief complaints of lower abdominal pain and fever. The patient had history of recurrent urinary tract infection for last 8 years. He was recently treated for urinary tract infection caused by* Pseudomonas aeruginosa *and was catheterized for last 2 weeks. After the necessary physical examination, the patient was suggested for laboratory investigation. In urine microscopic examination, significant bacteriuria with large numbers of pus cells per low power field was detected.

On the basis of the patient's clinical symptoms, history of recurrent urinary tract infection, and urine microscopic examination report, the patient was empirically treated with ofloxacin. The abnormal laboratory findings seen were increased C-reactive protein (15 mg/dL), raised blood creatinine (2.7 mg/dL), and raised total leukocyte count (17,000 cells/mm^3^) with neutrophilia (80%).

**Figure 1:**
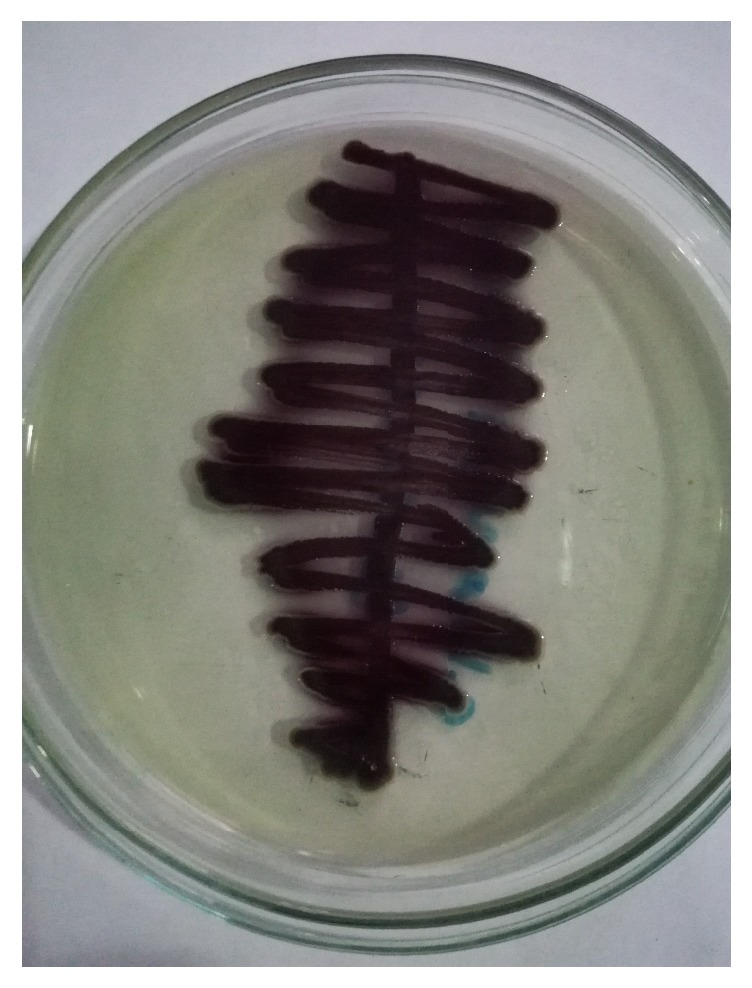
Colonies of* Chromobacterium violaceum* in cystine lactose electrolyte deficient agar.

The clean catch urine plated on cystine lactose electrolyte deficient agar showed the growth of single type of bacterial colonies in the concentration more than 10^5^ cfu/mL after 24 hrs of aerobic incubation at 37°C (Figure 1). The colonies grown were around 2 to 3 mm in diameter, convex, nonlactose fermenting, round, glistening, and smooth and produced violet nondiffusible pigment (Figure 1). The organism was Gram-negative rod and was motile and catalase and oxidase producing, citrate utilizing, and nitrate reducing. Further, the bacterial isolate decarboxylated arginine but did not decarboxylate lysine and ornithine and hydrolysed gelatin but did not hydrolyse esculin. It did not produce urease, indole, and DNase. Methyl red test and Voges-Proskauer test were negative. In triple sugar iron agar, it gave alkaline slant by acidic butt without any gas and hydrogen sulfide. The bacterium utilized glucose, fructose, and trehalose but did not utilize mannitol, sucrose, lactose, and xylose. The pigment formation was observed only after incubation under aerobic condition.

On the basis of all these properties, the organism was identified as* Chromobacterium violaceum*. For antimicrobial susceptibility testing, Kirby-Bauer disc diffusion technique was used. The organism was found to be sensitive toward ofloxacin, co-trimoxazole, amikacin, imipenem, norfloxacin, levofloxacin, and piperacillin/tazobactam and resistant toward nitrofurantoin and amoxicillin/clavulanic acid. Due to high propensity of the organism for hematogenous dissemination, blood culture was performed to check its presence in blood, which was negative. As the organism was found to be susceptible to ofloxacin, it was continued for one week. Finally, the repeat urine culture was performed to ensure the treatment success and the urine culture did not show any growth.

## 3. Case 2 (Bacteremia)

A 22-year-old male road traffic accident patient was admitted to the intensive care unit of a hospital in Kathmandu, Nepal, after receiving treatment for one week with no improvement in another hospital. The patient had multiple fractures with abrasion all over the body. The patient was serious but stable and had high body temperature. So, the necessary laboratory investigations were requested and the patient was put on meropenem, teicoplanin, and polymyxin B to cover all the bacterial infections including the nosocomial infections (if present), as the patient was transferred from another hospital.

**Figure 2:**
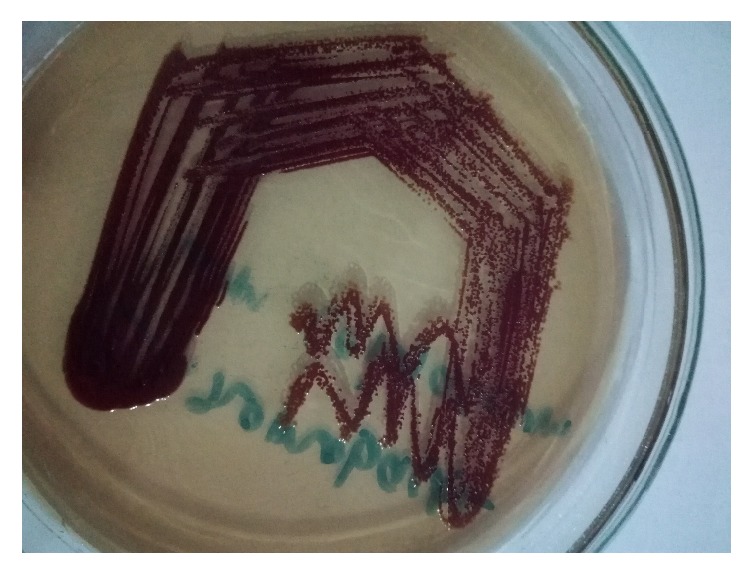
Colonies of* Chromobacterium violaceum* in MacConkey agar.

There were no clinical and radiological evidences of visceral abscesses. The abnormal reports included low hemoglobin (9.6 gms/dL), leucocytosis (12250 cells/mm^3^) with neutrophilia (89%) and lymphocytopenia (4.2%), low packed cell volume (30.7%), and low red blood cells count (3.54 millions/mm^3^). The blood culture performed in BACTEC 9050 showed the growth of bacterial pathogen, which on further identification by phenotypic methods (as performed in case 1) was confirmed to be* Chromobacterium violaceum* (Figure 2). The antimicrobial susceptibility testing performed by Kirby-Bauer disc diffusion technique showed that the organism was susceptible to nalidixic acid, ofloxacin, meropenem, tigecycline, chloramphenicol, and gentamicin but resistant to ceftriaxone, cefepime and polymyxin B. So, on the basis of the culture report received, the treatment with meropenem was continued for one week and the patient was again screened for any infections, if present, by performing bacteriological culture of different clinical samples and all were negative.

## 4. Discussion

In Nepal, recently, the infections caused by* Chromobacterium violaceum* have been increasingly reported [1, 3–5]. The reason for this may be either the increased rate of identification of the organism (due to development of sophisticated laboratory with skilled manpower) or the climate change [7]. Due to temperature sensitivity of this organism, the effect of global climate change has caused* Chromobacterium violaceum* to spread to wider geographical location [7, 8]. The organism has low virulence and is responsible for causing fatal sepsis in immunocompromised patients or in case of inappropriate antibiotic therapy [7]. In our case, although the patients were immunocompromised, they did not develop any complications due to timely proper treatment they received.

The* Chromobacterium violaceum* infections are acquired either through oral route by consumption of contaminated water or food or due to exposure of damaged skin to contaminated soil or water (mainly stagnant or recreational water) or during surgery [1, 6]. So, the road traffic accident patient might have got the infection through the abrasions present in his body, while chronic kidney disease patient might have got the infection due to urinary catheterization, which is also a risk factor for urinary tract infection. Similar to our study, Pant and Sharma [3] from Nepal and Ma et al. [9] from China reported the urinary tract infections caused by* Chromobacterium violaceum* in kidney disease patients. However, Pant et al. [1] from Nepal and Swain et al. [7] from India reported the urinary tract infections caused by this organism in immunocompetent adults. In accordance with our case, Parajuli et al. [5] reported a case of bacteremia caused by* Chromobacterium violaceum* from Nepal which they managed with timely proper treatment, while Ansari et al. [4] reported a case of fatal sepsis. Similarly, Mohan et al. reported a case of urinary tract infection caused by* C. violaceum* [10], while Ray et al. [11], Madi et al. [12], Karthik et al. [13], and Kar et al. [14] reported the cases of septicemia caused by the same organism in India. Further, Kaniyarakkal et al. [15] reported the cases of both urinary tract infection and septicemia from India.

*Chromobacterium violaceum* is known to be highly resistant to penicillins and cephalosporins but sensitive to carbapenems, fluoroquinolones, and aminoglycosides [7, 16]. In our study also the organism isolated from the case of urinary tract infection was found to be resistant toward amoxicillin/clavulanic acid and nitrofurantoin but sensitive toward ofloxacin, cotrimoxazole, amikacin, imipenem, norfloxacin, levofloxacin, and piperacillin/tazobactam. Similar types of drug susceptibility patterns were also reported by Pant et al. [1] and Pant and Sharma [3] in* Chromobacterium violaceum* isolated from two different cases of urinary tract infection. But, in contrast to our study, Swain et al. [7] reported the bacterium to be sensitive to nitrofurantoin; however, the susceptibility of the bacterium to other remaining antibiotics tested was similar to our study.

Similarly, in case of bacteremia patient the organism was susceptible to nalidixic acid, ofloxacin, meropenem, tigecycline, chloramphenicol, and gentamicin but resistant to ceftriaxone, cefepime, and polymyxin B. However, Parajuli et al. [5] reported the susceptibility of* C. violaceum* to cephalosporins, aminoglycosides, and fluoroquinolones and resistance to polymyxin B.

Because of high possibility of hematogenous dissemination to visceral organs and frequent relapse, long courses of antimicrobial therapies are recommended for treatment of infections caused by* Chromobacterium violaceum* [1, 17] but we did not find any clinical or laboratory or radiological evidences of hematogenous spread and visceral abscesses. So, the treatment was given only for one week but a suggestion to visit hospital as soon as any suspicious symptoms appear was given to the outpatient [1]. Similarly, the patient admitted to intensive care unit was put under strict observation for any suspicious symptoms.

As* Chromobacterium violaceum* has high tendency for hematogenous spreading causing sepsis which may cause death in as less as one week, the timely proper treatment is necessary to avoid the possible complications and hence to save patient's life [1]. In our case, the patients did not get any complications related to* Chromobacterium violaceum* infection due to timely proper treatment they received.

## 5. Conclusions

The infections caused by* Chromobacterium violaceum* may be treated successfully, if prompt proper treatment is started. For this, the clinicians should be aware of this rare but fatal infection. From Nepal, the infections caused by* Chromobacterium violaceum* have been increasingly reported.
